# Cardioneuroablation eliminating cardiac asystole associated with area postrema syndrome: a case report and literature review

**DOI:** 10.3389/fcvm.2024.1453166

**Published:** 2024-10-31

**Authors:** EnRun Wang, YuanJing Li, Gang Yu, Gang Liu, Jiang Deng, YanFei Wang, Wei Yang, GuoDong Chen, Dennis W. Zhu, FengPeng Jia

**Affiliations:** ^1^ Department of Cardiovascular Medicine, The First Affiliated Hospital of Chongqing Medical University, Chongqing, China; ^2^ Department of Neurology, The First Affiliated Hospital of Chongqing Medical University, Chongqing, China; ^3^ Cardiac Electro-Physiology Laboratories, Regions Hospital, St. Paul, MN, United States

**Keywords:** cardioneuroablation, asystole, NMOSD, medullary lesions, area postrema syndrome

## Abstract

**Background:**

There have been few instances of symptomatic bradycardia-arrhythmia in the context of area postrema syndrome (APS), and some of them have been implanted permanent pacemakers. Cardioneuroablation (CNA) has emerged as a viable therapy for the treatment of syncope induced by neutrally mediated bradycardia or atrioventricular block.

**Methods:**

We report a young patient with recurrent cardiac asystole and syncope following persistent hiccups caused by neuromyelitis optica spectrum disorder (NMOSD), who successfully completed CNA treatment and avoided permanent pacemaker placement. We also summarized and analyzed 20 previously reported cases that were relevant to APS with bradyarrhythmia.

**Results:**

In a patient with NMOSD, CNA can efficiently and safely eradicate symptomatic bradycardia-arrhythmia. A total of 21 cases were identified in the final analysis (including our case). The average age was 51 years old and female patients accounted for 38.1%. Brady-arrhythmia was presented in all patients, and 9 patients were implanted temporary or permanent pacemakers. 4 of the 9 patients were received permanent pacing therapy because they were not weaned off pacing support after etiological treatment.

**Conclusions:**

Cardiac asystole and syncope after persistent hiccups may be the first signs of APS of medullary lesions, and CNA may be a useful therapy option for these patients in experienced centers. We believe that in this scenario, CNA may be a superior therapeutic option than permanent pacemaker placement. Additionally, the statement also serves as a cautionary reminder for health care professionals to establish an association between bradyarrhythmia and APS of medullary lesions in their clinical practice.

## 1 Introduction

The immune-mediated condition neuromyelitis optica spectrum disorder (NMOSD) is characterized by acute demyelinating and/or necrotizing lesions in the optic nerves and/or spinal cord. As a disease with medullary lesions, NMOSD manifests in a wide range of ways, from common ocular neuritis, acute myelitis, and area postrema syndrome (APS, such as hiccups, nausea, and vomiting) to more usual symptoms including syncope and cardiac arrhythmias. Some of those patients with bradyarrhythmias received temporary or permanent pacemaker implantation. Aquaporin 4 (AQP4) is a water channel expressed in “Astrocytes”, and anti-AQP4 antibody is a specific marker for NMOSD (1, 2).

Cardioneuroablation (CNA) targeting the cardiac parasympathetic ganglionated plexus (GP) has emerged as a viable treatment for syncope induced by neutrally mediated bradycardia or atrioventricular block in recent years (3). As a result, we thought this unique method may be effective in treating cardiac asystole induced by medullary-related neurologic disease such as NMOSD in this case. We present the case of a young woman who had persistent hiccups followed by repeated syncope owing to extended asystole pauses and was effectively treated with CNA. We also performed a systematic review of the literature on the management of symptomatic bradycardia-arrhythmia in patients with NMOSD or other Medullary diseases.

## 2 Case report

**Figure 1:**
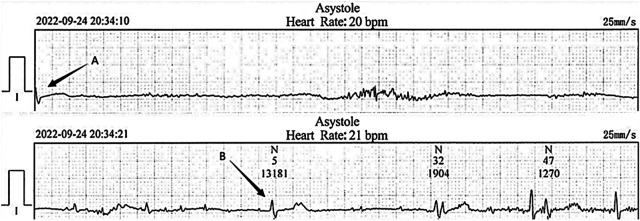
Electrocardiogram recorded at the local hospital. The cardiac arrest duration was 13.1 s following severe hiccups (between arrow **A** and **B**).

The patient was a 34-year-old Chinese female with a history of hypothyroidism and systemic lupus erythematosus (SLE) for 3 years. She took a minimal maintenance dose of steroids and 50 micrograms of levothyroxine sodium per day. Her SLE was in stable condition and thyroid function test kept normal during routine follow-up. 10 days before admission when she developed hiccups, nausea and vomiting without obvious causes. While visiting a nearby clinic, metoclopramide and intravenous fluids were ordered. However, the problems persisted. Five days earlier, she was admitted to a local hospital for repeated episodes of sudden onset dizziness and syncope following bouts of hiccups. She underwent a head computed tomography (CT) scan and without any positive findings. A 24-h Holter-monitor disclosed episodes of profound sinus bradycardia with slowest heart rate down to 14 bpm. There were 297 times of R-R intervals >2.0 s with the longest pause of 13.1 s (Figure 1). Permanent pacemaker therapy was offered but declined by the patient. After transferring to our cardiovascular department, the patient continued to have hiccups, nausea, and syncope without decreased vision and limb weakness. Routine laboratory examinations and markers of myocardial injury were in normal limitation. Thyroid function test was normal. Cardiac ultrasonography showed no abnormalities. Her consciousness was intact, and there was no abnormality in the cranial and peripheral nerves. A temporary pacemaker was implanted to maintain normal heart rate and prevent further syncope.

**Figure 2:**
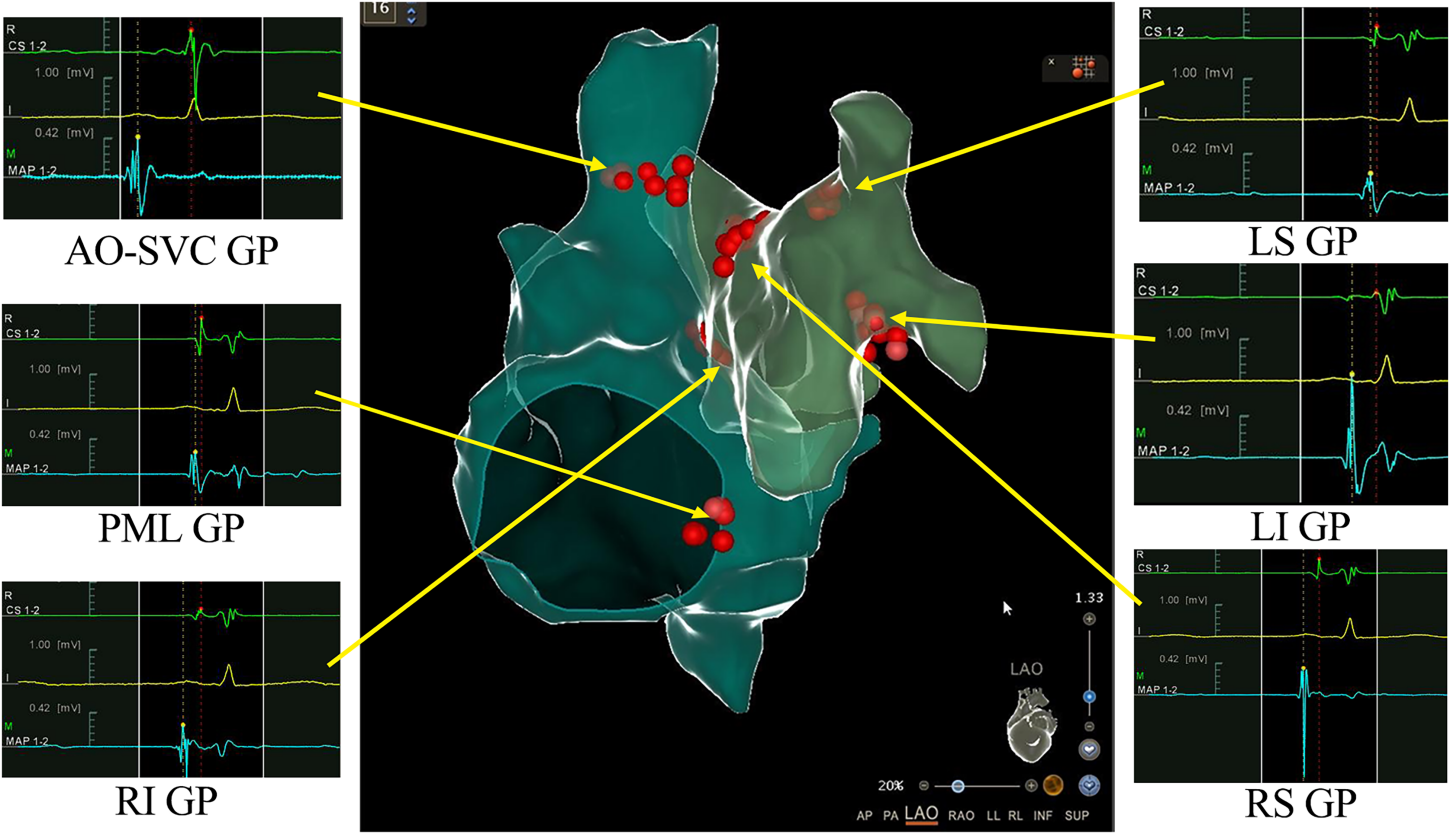
3D view of ganglionated plexus (GPs) groups distribution and ablation in the right and left atrium. Combined 3-dimensional electroanatomic mapping and radiofrequency (RF) ablation based on presence of fragmented/fractionated potentials in sinus rhythm. Red dots indicate RF application on the GPs sites. AO-SVC GP, aorto-superior vena cava GP; PML GP, posteromedial left GP; LS GP, left superior GP; LI GP, left inferior GP; RS GP, right superior GP; RI GP, right inferior GP.

**Figure 3:**
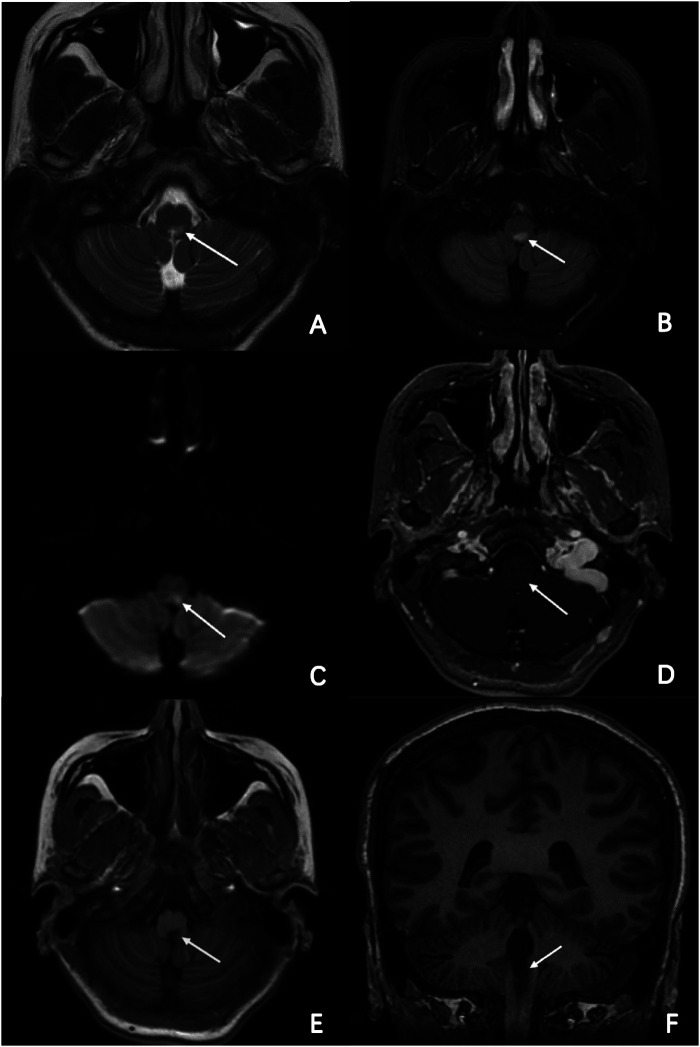
MR imaging of the brain and part of cervical cord. Axial T2-weighted, FLAIR and DWI brain MRI revealed a high-intensity lesion in the dorsal part of the medulla oblongata and cervical cord. **(A–C**; arrowheads**)** Axial enhanced T1-weighted and normal T1-weighted brain MRI revealed a low-intensity lesion in the same segment, **(D,E**; arrowheads**)** and clearer in coronal view. (**F**; arrowheads).

The patient was consulted by the neurology department at the very beginning and NMOSD was suspected. But considering that her symptoms and signs were not typical, we finally decided not to use methylprednisolone before her head MRI test done. However, head and neck MRI could not be performed because implanted temporary pacemaker and the lead were non-MR-conditional. After detailed discussion and counseling, patient and family agreed to proceed with CNA. The procedure was performed under Moderate sedation and routine electrophysiological testing was completed. The ganglionated plexus groups were mapped and ablated using a 3D electroanatomic mapping system, guided by anatomy and fragmented potentials. (CARTO3 Version 6; Biosense Webster, Diamond Bar, CA, USA) and a Thermocool® SmartTouch (Biosense Webster, Diamond Bar, CA, USA) irrigation catheter. The right atrium was initially reconstructed. Local fragmented intra-cardiac electrograms in the areas of aorto-superior vena cava GP and posteromedial left GP were eliminated (Figure 2). The left atrium was reconstructed and fragmented potentials were mapped. Ablation delivered at the targeted GPs (left superior GP, left inferior GP, right superior GP, and right inferior GP) (Figure 2). After ablation, the resting sinus rate increased from 54 bpm to 68 bpm, the long R-R interval > 2.0 s following hiccups was no longer present and temporary pacemaker was removed. There was no major complication related to the procedure. Two days after CNA procedure, persistent hiccup, vomit, and anorexia were still present and refractory to conventional medical therapy, but the episodes of dizziness and syncope had completely resolved. A 48-h Holter monitoring confirmed no R-R interval >2.0 s. MRI of the skull and spinal cord revealed a lesion at dorsal medulla oblongata (Figure 3). As the cerebrospinal fluid was tested positive for anti-AQP4 antibody, she was diagnosed as NMOSD. Considering the patient's low BMI (19.3) and the need for anti-coagulation therapy after CNA, methylprednisolone pulse therapy (800 mg per day instead of 1,000 mg) was given for five days and then changed to oral prednisolone with simultaneously ofatumumab for sequential immunosuppressive therapy. Her symptoms resolved and was discharged five days later with prednisolone (45 mg per day for the first 2 weeks, then gradually reducing dosage under the supervision of a physician), levothyroxine sodium 50 Ug per day and Edoxaban 30 mg per day for 2 months.

She remained asymptomatic and 24-h Holter monitor demonstrated normal sinus rhythm with appropriate chronotropic response at 3- and 6-months follow-up.

## 3 Discussion

Area Postrema Syndrome (APS) is rare among the symptoms of NMOSD, presenting as intractable nausea, vomiting, and hiccups, this condition is commonly misdiagnosed clinically. Herein, we described a NMOSD patient with dorsal medulla oblongata involvement who initially presented with persistent hiccups, nausea, vomiting, and episodes of syncope due to cardiac asystole. Neuromyelitis optica frequently affects the medullary tegmentum, leading to persistent hiccups and nausea in patients (4). The differential diagnosis for persistent hiccups includes central nervous system (CNS) diseases, gastrointestinal, thoracic, cardiac, toxic-metabolic and drug-related disorders (5) In most cases, the mechanism of hiccup is involved in the following 3 pathways: (1) the afferent pathway of the “vagal nerve, the phrenic nerve, or the thoracic-sympathetic nerve”, (2) the medulla oblongata, including the respiratory center, the “ambiguous nucleus”, and the “reticular formation”, and (3) the efferent pathway of the phrenic nerve or the motor nerves to respiratory muscles (6). Previous research revealed that parasympathetic stimulation and sympathetic inhibition caused by the lesion of the solitary nuclei in medulla oblongata can result in bradycardia and asystole (7). We assumed that lesions in medulla oblongata, specifically the solitary nucleus and medullary tegmentum triggered hiccups and bradycardia. Hiccups may further enhance parasympathetic outflow and result into severe bradycardia and long pauses.

When it comes to NMOSD with bradyarrhythmia, it is necessary to make a differential diagnosis with other neurological diseases such as multiple sclerosis and infarction, which can also give rise to cardio-inhibitory arrhythmia. The published studies are limited and the most of them are case reports or observation of small series. We summarized the published reports of bradycardia arrhythmia associated with the medullary lesion in Table 1 (7–26). A total of 21 cases were identified in the final analysis (including our case). The average age was 51 years old and female patients accounted for 38.1% (8/21). Brady-arrhythmia was presented in all patients, in addition, one case was combined with ventricle tachycardia and another one with paroxysmal atrial fibrillation, respectively reported by Berry and Koay (15, 21). Symptomatic bradycardia arrhythmia was secondary to NMOSD (9 cases), medullary infarction (8 cases) and multiple sclerosis (4 cases) in sequence. Notably, most of the patients with bradyarrhythmia had lesions in the medulla oblongata, except one patient with cervical cord lesions, one patient associated with midbrain and another case without MRI results.10-12 9 patients were received pacing therapy. 4 of the 9 patients were implanted permanent pacemaker because they were not weaned off pacing support after etiological treatment. Interestingly, none of MS patients had pacing treatment but three of four patients had noticeable improvement. Most of patients had better outcomes after pacing support and treatment of the underlying disease except one death (19).

**Table 1:** Summary of published case reports of bradycardia-arrhythmia associated with the medullary lesion.

Reference	Age/sex	Cardiac manifestations	Medullary lesion on MRI	Etiology	Treatment for ARS	Result
Bigi et al. (8)	16y/M	Sinus bradycardia	Area postrema	NMOSD	–	Recovery
Okada et al. (9)	78y/M	Cardiorespiratory arrest, syncope for twice	Medulla oblongata to C2, C3/4-C4	NMOSD	–	Partially improved, still unable to walk
Zhou et al. (10)	44y/M	Recurrent syncope	Dorsal medulla oblongata, hypothalamus, aqueduct of midbrain	NMOSD	–	Recovery
Komaki et al. (11)	77y/M	Sinus arrest, syncope for once	Area postrema	NMOSD	TPM	Improved, TPM removed after therapy, no syncope
Endo et al. (12)	22y/F	Sinus arrest, syncope for once	Dorsal medulla oblongata	NMOSD	TPM	Markedly improved, TPM removed before therapy, no syncope
Tsouris et al. (13)	42y/M	Sinus arrest, recurrent syncope	Lower part of the medulla oblongata (dorsal vagal complex), optic nerve	NMOSD	PPM	Recovery
Hamaguchi et al. (14)	77y/F	Sinus arrest, recurrent syncope	Dorsal medulla oblongata, cervical cord	NMOSD	PPM	Partially improved, no syncope
Berry et al. (15)	53y/M	Sinus pauses, sinus bradycardia, ventricular tachyarrhythmia	Anterior third ventricular floor, fourth ventricular floor, hypothalamus, optic chiasm, T5/6 level of spinal cord	NMOSD	PPM	Recovery
Our patient	34y/F	Sinus arrest, recurrent syncope	Dorsal medulla oblongata, cervical cord	NMOSD	TPM, CNA	Markedly improved, TPM removed after therapy, no syncope
Funakawa et al. (16)	54y/F	Recurrent syncope	Upper cervical cord and C5-C6 region	Multiple sclerosis	–	Recovery
Sakakibara et al. (17)	31y/F	Orthostatic hypotension, recurrent syncope	Left posterior peduncle, left parietal area, caudal paramedian tegmentum and the base of the medulla	Multiple sclerosis	–	Improved
Jurić et al. (18)	20y/F	Sinus bradycardia	Left cerebellar hemisphere, and midbrain	Multiple sclerosis	–	Improved
Nagashima et al. (19)	64y/F	Sinus bradycardia	No MRI result	Multiple sclerosis	–	Died after 5y
von Heinemann et al. (20)	45y/M	Sinus arrest, recurrent syncope	Right dorsolateral medulla	Infarction	TPM,	Markedly improved, TPM removed after therapy, no syncope
Koay et al. (21)	52y/M	Paroxysmal atrial fibrillation, sinus arrest	Left lateral medulla, left medial cerebellar hemisphere	Infarction	PPM	Markedly improved
Alloush et al. (22)	64y/M	Recurrent syncope, sinus arrest, sinus bradycardia	Left-sided dorsal lateral medulla	Infarction	PPM	Recovery
Mesquita et al. (23)	50y/M	Recurrent syncope	Inferior lateral right medulla oblongata	Infarction	–	Recovery
Takazawa et al. (7)	78y/F	Sinus arrest, cough syncope, hypotension	Bilateral medial regions and right tegmentum	Infarction	–	Improved
Lee et al. (24)	55y/M	Recurrent syncope, sinus arrest, sinus bradycardia	Left lower lateral medulla oblongata	Infarction	–	Markedly improved
Huynh et al. (25)	67y/M	Pre-syncope	Right-sided dorsolateral medulla	Infarction	–	Improved
Gofton et al. (26)	60y/M	Syncope, sinus bradycardia	Right-sided lateral medullary infarction	Infarction	–	Improved

PPM, permanent pacemaker implantation; TPM, temporary pacemaker implantation; CNA, cardioneuroablation; ARS, arrhythmias.

The anatomical lesion sites of these cases correlated with varied clinical presentations in neurological disorders that impact cardiovascular systems. Lesions in the medulla oblongata, particularly around the area postrema, could cause conducted disruptions manifesting as syncope and arrhythmias (8, 11, 13, 14). Lesions extended to cervical segments in certain cases,which would lead to a broader range of autonomic dysfunction, presenting as severe cardiac symptoms like sinus arrest (9). The spread of lesions to lateral or inferior parts of the brainstem or spinal cord could result in cardiogenic shock. This heterogeneity in lesion sites illustrates the intricate interactions between neuroanatomy and cardiovascular systems, necessitating tailored diagnostic and therapeutic approaches for optimal management of these conditions.

Although pacemaker is effective for treatment of symptomatic bradycardia-arrhythmia in patients with NMOSD, an implanted non-MR-conditional pacemaker may complicate the MR test which plays a key role in the earlier diagnosis of the disease. Pacemaker implantation itself is associated with complications such as infection, leads breakage and leads dislodgement (twiddler syndrome) (27). The bradycardia-arrhythmia in most patients with NMOSD is temporary and reversible when the primary disease is appropriately managed. CNA has been used to treat vasovagal syncope from functional atrioventricular block and/or sinus bradycardia in the recent years. Majority of the literature suggested the technique may be safe and effective in selected population (28). In our case, the profound cardiac asystole was likely mediated through the intrinsic cardiac autonomic nervous system. Catheter ablation of cardiac GPs may represent a better option than a permanent pacemaker insertion. To the best of our knowledge, this is the first instance of CNA successfully and safely removing symptomatic bradycardia arrhythmia in a patient with NMOSD. More research is needed to corroborate our findings.

Several limitations of our study should be acknowledged. There are limited studies in the literature on the treatment of bradycardia-arrhythmia in individuals with medullary disorders. The long-term efficacy of CNA in this specific disease needs to be confirmed in larger population and by registries. Although bi-atrial ablation procedure in this case achieved good outcome, right or left atrial ablation alone may be sufficient as ablating different GP sites may have different effects on sinoatrial and atrioventricular nodal function (29). Cost-effectiveness should be considered in the clinical practice.

## 4 Conclusions

This case demonstrated that medullary lesions may generate substantial bradycardia and asystole as the initial presentation of NMOSD before the onset of distinct neurological signs. The use of CNA may be more beneficial than implanting a pacemaker permanently. Additionally, the statement also serves as a cautionary reminder for health care professionals to establish an association between bradyarrhythmia and NMOSD in their clinical practice.

## Data Availability

The original contributions presented in the study are included in the article/Supplementary Material, further inquiries can be directed to the corresponding author.
